# The Analysis of Fuzzy Qualitative Comparison Method and Multiple Case Study of Entrepreneurial Environment and Entrepreneur Psychology for Startups—Evidence From Guangdong-Hong Kong-Macao Greater Bay Area and Southeast Asia

**DOI:** 10.3389/fpsyg.2021.751309

**Published:** 2022-01-25

**Authors:** Chien-Chi Chu, Zhi-Hang Zhou, Xin Wang, Haichao Wu, Yue Tian, Zepai Cai

**Affiliations:** ^1^Business School, Foshan University, Foshan, China; ^2^Guangdong University of Technology, Guangzhou, China

**Keywords:** startups, entrepreneurial environment, entrepreneur psychology, fsQCA, multiple case study, Guangdong-Hong Kong-Macao Greater Bay Area, Southeast Asia

## Abstract

Recently, scholars have begun to shift their focus toward the idea of the marketization of startups and the relationship with entrepreneurial psychology or other factors; however, the establishment of a unified and clear standard of entrepreneurship educational methods remains unfulfilled. Our study investigates 46 representative startups in four industries, including financial technology, biotechnology, education, and cultural tourism areas in Guangdong-Hong Kong-Macao Greater Bay Area (GBA) and Southeast Asia (SEA) to observe factors from different backgrounds but matter in common for building entrepreneurship education systems and methods in different countries. We used the fuzzy qualitative comparison method (fsQCA) to survey startup entrepreneurs and executives through questionnaires, selecting startup key factors including entrepreneurial psychology (optimism, passion, self-efficacy), product advantage, market and cultural environment, entrepreneurial policy, and geographical advantage. The survey was conducted on six key variables, namely, geographical advantage, to observe the conditional grouping and paths of factors influencing the establishment of Startups from an overall perspective. This study explores the path combination that plays a key role in the establishment of new enterprises, and further uses specific industry cases to verify the rationality and credibility of the path combination. The main conclusions are as follows: (1) There are five combination paths affecting the establishment of new enterprises, which are “psychology and market,” “psychology, product, and region,” “psychology, culture and policy,” “psychology, market, and culture,” and “market, policy, and region” combination paths; (2) Entrepreneur psychology, market environment, and entrepreneurship policy are the core conditions to improve the effectiveness of the establishment of new enterprises, while the other three variables are non-core variables in different paths; (3) There are different paths of entrepreneurial paths and factor combinations in different regions or industries.

## Introduction

How to help startups survive and grow while dealing with different environments is the greatest challenge for entrepreneurs who need to make sound decisions in the face of various unknown situations, maintain a stable mindset, and actively adapt to the external environment of the business to ensure its survival (Sarasvathy, 2001; McMullen and Shepherd, 2006).

An increasing number of scholars have begun to focus on the fact that the combination of entrepreneurial psychology and entrepreneurship research can enrich and improve the current theoretical framework of entrepreneurship (Frese and Gielnik, 2014), and some researchers have addressed the relationship between entrepreneurial psychology and entrepreneurial motivation in the transportation industry (Shi and Wang, 2021), while other researchers have added the psychology of entrepreneurship in college students to the study of entrepreneurial cognitive interaction (Liu and Yu, 2021). Therefore, this study first selects the positive elements of entrepreneurial psychology as the first element that shapes the process of establishing a new venture, which includes optimism, passion, and self-efficacy, all of which have been demonstrated empirically by research identifying their exact impact on entrepreneurial behavior and decision-making (Prandelli et al., 2016; Xie et al., 2016; Gu et al., 2019).

The entrepreneurial environment is a complex context involving multiple factors, and to link it theoretically with entrepreneurial intentions, domestic and foreign scholars have extensively researched it. By examining the existing literature, it is found that the current mainstream view divides the entrepreneurial environment into two aspects: the internal environment and the external environment. The internal environment refers to the entrepreneur’s attributes and capabilities, while the external environment refers to the market environment, cultural environment, policy environment, geographical environment, and other factors faced by the startups (Fogel, 2001).

The main problems in the current research community are the following: 1) most researchers have only studied the correlation between a single psychological factor and entrepreneurship, and few scholars have studied the combined effect of multiple psychological factors; 2) most researchers study the impact of a single environmental variable on entrepreneurship research, ignoring the direct synergy and interaction of multiple variables; and 3) current research mostly studies the entrepreneurship phenomenon in a certain industry in a certain place, and lack comparative studies across regions and industries.

Due to the shortcomings of the above research on the influencing factors of entrepreneurship, the influencing factors of new ventures are currently divided into psychological factors, the entrepreneurial environment, and other factors. As a result, the current research is limited to clarifying the roles of a small number of influencing factors on entrepreneurship and lacks the linkage and combination effect analysis of multiple factors. This study seeks first to apply the fuzzy qualitative comparison method (fsQCA) method to analyze the path of the entrepreneurial process under a variety of influencing factors in combination, using a case analysis method to confirm different industries in different regions and the feasibility and the rationality of this path, and further to include some defects and deficiencies in fsQCA to complement and expand the research field.

The innovation of this article mainly lies in its combination of different research perspectives. In the past, scholars usually analyzed data from a single case or a single industry, leading to a lack of multilocation studies conducting cross-industry factor analyses. Against the background of an “area” initiative, Guangdong-Hong Kong-Macao Greater Bay Area (GBA), and Southeast Asian countries exhibit commonalities and differences in entrepreneurial factors thus have profound research value. Therefore, this paper explores the influence of different industrial entrepreneurship factors in these countries and regions from a cross-regional perspective, which holds important theoretical significance for the joint development and interaction between the GBA and southeast Asian enterprises.

A method capable of achieving this aim, fsQCA is a qualitative analysis method of analysis that explores the effects of multiple factor combinations and has been applied by several researchers in recent years to examine, for example, firm performance (Kraus et al., 2018). Therefore, this study uses fsQCA to combine three psychological factors (optimism, passion, and self-efficacy) as one of the founding factors of startups, addressing the issue of the entrepreneurial environment by selecting product advantage as an internal environment variable and market environment, cultural environment, entrepreneurial policy, and geographical advantage as external environment variables, and then applies the analysis to select startups in four industries in GBA and Southeast Asia for comparison. The fsQCA method is used to find the different factor path combinations of new startups in each region, and to explore the commonalities and differences in the factor path combinations of new startups in different regions and industries.

## Literature Review

### Entrepreneurial Psychological Impact on Startups

The key point in studying the psychological efficacy aspects of entrepreneurs is to choose definite directional indicators and methods that can quantify the research object, and the current indicators in studying the psychological direction of entrepreneurs are generally optimism, entrepreneurial passion, and self-efficacy (Zhang et al., 2019).

Optimism as a positive mindset (Carver et al., 2010) reflects the extent to which people generally hold favorable expectations about their future, and when caught in a difficult situation or facing a crisis, optimism can help a person avoid pessimism and a downward spiral. With the increase in research on the combination of optimism and entrepreneurship, the psychology of entrepreneurial optimism has gradually gained the interest of scholars; however, the focus of the current research is limited to the relevance of entrepreneurial optimism to the performance of startups (Liu et al., 2016) or concerns the moderating role of corporate entrepreneurial competencies (Fang et al., 2011) and rarely involves the association with the elements of startup establishment, exploring its impact on the process of business formation as an element of the pathway portfolio.

Entrepreneurial passion, as a strong emotion in individuals, can be a powerful driver of behavior (Cardon et al., 2017). Entrepreneurial passion can be classified as a passion for growth, passion for people, passion for products or services, passion for inventions, passion for competition, and passion for social causes. Entrepreneurial passion comes from the entrepreneur’s assessment of the future state of the business, which motivates the entrepreneur to solve the dilemmas faced in the entrepreneurial process and provides the entrepreneurial venture with a strong and lasting key driver who will stay engaged at all times during the entrepreneurial process. In early studies of entrepreneurial passion, it was mostly explored in terms of its effects on an individual’s abilities, such as persistence, effort, and perseverance when encountering difficulties (Smilor, 1997), as well as in terms of the sociological effects of passion on individual behavior (Mette, 1999). Studies have expanded the focus on entrepreneurial passion to entrepreneurial ideas, entrepreneurial opportunities, and entrepreneurial firm performance (Biraglia and Kadile, 2017; Shaver and Davis, 2017; Newman et al., 2021). This study builds on this foundation to explore the relationship between entrepreneurial passion and the conditions required for the establishment of a startup business.

Self-efficacy is an optimistic belief about one’s ability to accomplish tasks in unknown situations. Self-efficacy is largely involved in the regulation of attention processes and the modeling of experiences, and it affects one’s work efficiency, decision making, and other events in a variety of ways. Research has suggested that self-efficacy can help individuals improve their perseverance and persistence in the face of unknown difficulties, and increase their positive perception of themselves (Bandura, 1999). Some researchers have suggested that the self-efficacy scale can be used to test individuals (Ding et al., 2009) in order to explore the relationship between entrepreneurial self-efficacy and entrepreneurial intention.

At the same time, in the context of economic globalization, the public’s cognition of entrepreneurship from the perspective of educational psychology affects national development strategies. Therefore, the interaction between educational psychology and entrepreneurship has also been studied by many scholars. Some scholars have studied the *status quo* of entrepreneurship education in colleges and universities through questionnaires and interviews. Only a very few students have clear seriousness and enthusiasm for entrepreneurship (Liu and Yu, 2021). There are also studies of the effect of entrepreneurship education in universities using online and offline survey methods. Researchers have found that the low proportion of college students choosing entrepreneurship is mainly due to the lack of entrepreneurship teachers and course planning (Sun, 2020). With the increase in studies of the interaction between educational psychology and entrepreneurship, scholars have begun to explore the impact of entrepreneurship on the performance of new ventures when college students were taken as the research object. The results showed that college students with higher education had higher integrity and entrepreneurship, which had a positive impact on the performance of enterprises (Zhang et al., 2021). Given the lack of entrepreneurship education from the perspective of educational psychology, many scholars have proposed solutions, such as building a value judgment system for college students (Chen et al., 2021) and strengthening the cultivation of cultural diversity (Li Z. J. et al., 2021). In addition, many researchers from the direction of educational psychology, the entrepreneur and employee characteristics of the empirical analysis and comparison (Yuan et al., 2020; Yuan and Wu, 2020), cover a number of promising industries such as instant messaging and barrier-free transportation (Wu et al., 2020; Yuan et al., 2020).

### Impact of the Entrepreneurial Environment on Startups

In the process of entrepreneurship, any business needs to rely on a certain entrepreneurial environment. Good entrepreneurial culture and entrepreneurial environment can largely influence the process of setting up a new startup business. The entrepreneurial environment is a set of external conditions that entrepreneurs have to adapt to Morgan (2011), essentially consisting of an institutional environment that covers three dimensions: normative, regulatory, and cognitive (Chien-Chi et al., 2020). The cognitive system involves people’s access to information and ability, which is part of the entrepreneur’s own ability, and the regulatory system includes laws, institutions, regulations, and government support, and restrictive behaviors (Gao and Duan, 2021), which can be defined as government policies.

In recent years, some scholars have researched the entrepreneurial environment and divided the institutional environment into three dimensions: normative, regulatory, and cognitive systems. However, there is also another view that distinguishes the entrepreneurial environment along three dimensions: institutional, market, and cultural, and it is argued that a good market environment can provide diversified development channels for entrepreneurs, which is important for the survival and expansion of enterprises (Li et al., 2020).

Based on the above studies, researchers have conducted research on the entrepreneurial process in both entrepreneurial psychology and the entrepreneurial environment, which are based on one variable, and few have studied the combination of factors in the process of new venture establishment; most have not focused on the influence of regional advantage on the entrepreneurial process or the linkage between regional advantage and other influencing factors (Asheim et al., 2007). However, the internal environment is as important as the external entrepreneurial environment, and product advantage (Langerak et al., 2004) is important for the survival and development of startups as a manifestation of a firm’s competitiveness in the market. In this study, as few researchers have studied the impact of multiple conditions on the process of startup establishment, it was decided to use fsQCA to investigate the impact of a combination of six main influencing conditions: entrepreneurial psychology (EP) (comprising optimism, entrepreneurial passion, and self-efficacy), product advantage (PA), market environment (ME), cultural environment (CE), entrepreneurial policy (EPY), and geographical advantage (GA), on startup establishment. Different grouping paths are expected to exist for new startups to reach the conditions of company formation in GBA and Southeast Asia due to their different locations, economic environments, and market environments.

### Application of Fuzzy Qualitative Comparison Method

As a popular path analysis method, fsQCA has been widely used in the field of entrepreneurship. At the micro-level, some scholars have used fsQCA to study the impact of time pressure on employees’ work enthusiasm (Yi et al., 2018), while others have explored the difference in the impact of different paths on the entrepreneurial activity of science and technology personnel by collecting data from 27 provinces and cities in China (Cui and Wu, 2021).

At the macro level, some researchers have conducted path analyses of the incubation performance factors and breakout paths of business incubators for science and technology enterprises, concluding that resources, industry, innovation, and comprehensive drive are concluded as four entrepreneurial drive paths (Zhao et al., 2021). Research has also found that four different paths may provide support for enterprises in mergers and acquisitions (Zhang et al., 2018). In terms of the influence of culture and economy on the development of the national entrepreneurship level, some scholars have conducted path studies on the relevant data of 44 countries, finding that confident culture and entrepreneurship were the main paths for the development of national entrepreneurship culture (Hu et al., 2020).

## Materials and Methods

### Research Methods

This study uses fsQCA to study this problem. fsQCA is a qualitative and quantitative method proposed by Ragin (2000) whose basic idea is to analyze the influence of the combination of multiple antecedent variables on the result variables with the help of the architecture theory and Boolean operation. To explain the complex causal mechanism behind the phenomenon, this research uses fsQCA in place of traditional regression analysis and factor analysis for the following reasons: First, new ventures, in addition to the psychological factors, should comprehensively consider the factors enterprises need to create while successfully adapting to these various factors, especially the environmental factors and objective factors; as the influence of the individual elements is very limited, we seek to use configurations that combine the effects of various factors to explain the cause of the newly established ventures.

Second, different regions have asymmetries in the establishment of new ventures; for example, it is argued that the entrepreneurship environment is a major cause of entrepreneurs having a high degree of activity, but there are also positive entrepreneurs in unsatisfactory entrepreneurial environment. Thus, using the fsQCA method to study the creation of elements of new ventures helps explore the combination of a variety of entrepreneurial paths and avoiding the asymmetry of elements.

Finally, compared with factor analysis methods, fsQCA has a low degree of data loss in the research process and low requirements for data integrity. In the study of small- and medium-sized samples, if the factor analysis method is used, data may be lost in the data calculation process, causing the final results to lack relevance and practicality. fsQCA is good for cross-case comparisons of small- and medium-sized samples and can distinguish the consistency and coverage of elements, as well as determine the synergies between elements. In conclusion, fsQCA is useful for solving configuration problems with multiple independent variables and can better meet the requirements of causal complexity and synergistic effects. It is also conducive to exploring multiple paths in the process of new enterprises and finding the essence and necessary conditions for the establishment of new enterprises.

### Variable Selection

In terms of variable selection, all 30 questions in the EP section were scored on a 5-level Likert scale; the language of the questionnaire was devised to be concise and easy to understand to improve readability for the respondents. The questionnaire was introduced as follows to participants: “Before completing the questionnaire, it should be noted that all the results of the questionnaire are for academic research only and do not involve the core issues of the company’s operation. Please complete the questionnaire truthfully.”

For the questionnaire on entrepreneurs’ psychology, we adapted the Life Orientation Test (LOt-R) modified by Herzberg et al. (2006) with a total of five statements, such as “can you expect the best result in most cases?,” “Passion is the core of entrepreneurship” (Cardon et al., 2005), and “Entrepreneurial passion can stimulate motivation.” Regarding the key driving force of entrepreneurship, the questionnaire followed one study (Cardon et al., 2013) regarding the three influencing factors of entrepreneurial passion: creation, discovery, and development, to develop a six-item questionnaire to test an individual’s entrepreneurial passion. To measure self-efficacy, the belief that one can cope with various pressures or challenging requirements, the questionnaire adapted the Self-Efficacy Scale (Luszczynska et al., 2005) for four items for scoring, such as “Am I sufficiently thoughtful and able to deal with unforeseen changes?” For product advantages (Sheng and Zhang, 2009), there are five items from the Improved Product Questionnaire, which has good reliability and validity; in terms of market environment, it refers to the analysis of the urban business environment (Li M. et al., 2021), including six items, and there is a motivational relationship between entrepreneurial cultural factors and entrepreneurial motivation of entrepreneurs. For culture, there are four questions about innovation cognition and entrepreneurial motivation factors in entrepreneurial motivation (Zhong et al., 2012). For entrepreneurship policy, respondents were asked a total of five questions about their satisfaction with local entrepreneurship policies, and for geographical advantage, the respondents responded to five questions about the convenience of transportation, transportation, and imports and exports.

### Data Collection

The questionnaire used in our survey consists of six subscales, EP, PA, ME, CE, EPY, GA, and is targeted at startup founders and executives. Before the questionnaire was distributed electronically online, a preliminary version was tested with five executives, who reported no problems understanding the questions. A total of 92 responses were collected from executives and founders of 46 startups in four industries in Guangdong, Hong Kong, Macao, and Southeast Asia. Six of the incomplete responses were discarded, leaving a total of 86 valid responses.

## Procedure

### Calibration Date

Since the data collected by the questionnaire in this study are not two-dimensional, it is difficult to apply fsQCA to them. Because fuzziness needs to be introduced to calibrate the questionnaire results and bring them into conformity with the measurement standards, this study uses the method of calibrating fuzzy membership to process the data. First, three anchor points need to be selected: full membership, an intermediate point, and no membership. Here, we follow Fiss (2011) and adopted three anchor points, 1, 3, and 5, corresponding to the three points 0.05, 0.5, and 0.95, respectively, in the Calibrate function. In addition, following Ragin (2006), this study avoided membership degrees of 0.5, calibrating such values to 0.49 or 0.51 according to the situation, thereby allowing suitable fuzzy values to be assigned to all the data. Moreover, to facilitate the subsequent comparison of paths for different countries and regions, the fuzzy data of different countries and regions were averaged, yielding Table 1. In addition, for the dependent variable of the new venture, we set the value between 0 and 1 according to the operating income, number of employees, size of the company, and other aspects of the enterprise, and used it as a measure of the difficulty of establishing a new venture.

**TABLE 1:** Fuzzy data mean value table.

	EP	PA	ME	CE	EPY	GA
GBA	0.91	0.74	0.57	0.53	0.57	0.82
Singapore	0.92	0.56	0.82	0.35	0.51	0.48
Philippines	0.38	0.47	0.65	0.52	0.61	0.58
Vietnam	0.74	0.46	0.68	0.59	0.54	0.27
Laos	0.29	0.35	0.52	0.53	0.29	0.70
Cambodia	0.37	0.48	0.68	0.43	0.71	0.64
Myanmar	0.43	0.21	0.56	0.32	0.57	0.71
Thailand	0.59	0.43	0.69	0.72	0.65	0.74
Malaysia	0.61	0.39	0.81	0.42	0.78	0.26
Brunei	0.56	0.41	0.60	0.76	0.35	0.63
Indonesia	0.72	0.38	0.86	0.74	0.52	0.34
East Timor	0.61	0.24	0.51	0.36	0.39	0.53

### Necessity Analysis

Unlike traditional regression analysis, fsQCA does not focus on the influence of a single variable on the result, but rather on the sufficiency or necessity of each scale item for the result variable. Therefore, before path analysis, we first performed a necessity analysis for each factor whose results are shown in Table 2.

**TABLE 2:** Necessity analysis table.

	Result			
Condition	High startup difficulty		Low startup difficulty	
	Consistency	Coverage	Consistency	Coverage
EP	0.854245	0.704138	0.495420	0.541055
∼EP	0.443218	0.398667	0.729092	0.868896
PA	0.782760	0.633789	0.545383	0.585073
∼PA	0.487545	0.447339	0.658632	0.800675
ME	0.739021	0.623499	0.521549	0.582997
∼ME	0.505736	0.443765	0.663183	0.771000
CE	0.790961	0.608555	0.573555	0.584671
∼CE	0.460183	0.448875	0.615997	0.796098
EPY	0.848037	0.653611	0.556644	0.568427
∼EPY	0.440050	0.428288	0.660791	0.852099
GA	0.834496	0.609327	0.619796	0.599608
∼GA	0.451649	0.472737	0.596174	0.826768

If the level of factor consistency exceeds 0.9, the factor is a necessary condition for the outcome variable; if the level of factor coverage exceeds 0.9, the factor is a sufficient condition for the outcome variable. If the figure of consistency and coverage does not exceed 0.9, this indicates that the variable is neither sufficient nor necessary variable, whether the entrepreneurial difficulty is high or low, so there is no single factor that has a dominant effect on it, but rather it is the result of multiple conditions acting together (Xu et al., 2020), and since the individual variables are weakly explanatory, the model is next analyzed for path analysis.

### Path Analysis

The fuzzified variables are input into the truth table analysis to derive the truth table of the factors of new venture establishment in GBA and Southeast Asia can be obtained. Next, we edit the frequency and threshold of the truth table (Ragin and Amoroso, 2011). Since this study is a small sample analysis with a sample size ranging from 1 to 150, after model analysis, the case truncation value was set as 1, samples were not deleted, and the consistency threshold was 0.7. When samples with consistency below 0.7 are deleted, the truth table in Table 3 is the result. The value 1 in the truth table represents a high level of the respective factor, and the value 0 represents a low level of this factor. After checking the data, it was found that there were no contradictory combinations, that is, cases where the same combination of conditions leads to different results, indicating that the subsequent analysis can be carried out.

**TABLE 3:** Truth table.

EP	PA	ME	CE	EPY	GA	Number	Entrepreneurial difficulty	Raw consist.
1	1	1	1	1	0	4	1	0.903379
1	1	1	1	1	1	4	1	0.903379
1	1	0	1	1	0	1	1	0.881281
1	1	0	1	1	1	1	1	0.881281
1	0	1	1	1	0	1	1	0.859773
1	0	1	1	1	1	1	1	0.859773
1	0	1	0	1	0	4	1	0.785768
1	0	1	0	1	1	4	1	0.785768
0	1	1	0	1	0	1	1	0.76194
0	1	1	0	1	1	1	1	0.76194
1	0	0	1	1	0	4	1	0.725892
1	0	0	1	1	1	4	1	0.725892
1	0	0	1	0	0	1	0	0.644628
1	0	0	1	0	1	1	0	0.644628
0	1	0	0	1	0	4	0	0.575056
0	1	0	0	1	1	4	0	0.575056
0	1	0	0	0	0	2	0	0.572628
0	1	0	0	0	1	2	0	0.572628
0	0	0	0	0	0	2	0	0.522852
0	0	0	0	0	1	2	0	0.522852
0	0	0	1	0	0	2	0	0.497224
0	0	0	1	0	1	2	0	0.497224

After the standardized analysis of the conditional combinations, three solutions can be derived: a complex solution, intermediate solution, and simple solution. This study refers to the method applied involved in Ragin (2009), mainly using the intermediate solution as a reference, and taking whether the condition exists in both the simple and intermediate solutions as a criterion for determining the core and edge conditions; if the simple solution also exists in the intermediate solution, the variable is in the core condition, and if the condition in the simple solution does not exist in the intermediate solution, the condition is in the edge condition. Table 4 shows the combination of start-up factors in Guangdong, Hong Kong, Macao, and Southeast Asia under different paths:

**TABLE 4:** Path combination table.

Elements	Path combination					
	1	2	3	4	5	6
EP	•	•	•	•	⊗	⊗
PA	▪	▪	⊗		▪	
ME			•	•	•	•
CE		▪	⊗	▪		▪
EPY	⊗		▪	▪	•	•
GA	•	•		⊗	•	•
Raw coverage	0.380905	0.537391	0.265451	0.330855	0.29869	0.283335
Unique coverage	0.0398049	0.1467	0.0449656	0.0673974	0.0388339	0.0247567
Consistency	0.855414	0.913966	0.786525	0.865006	0.770107	0.786469
Solution coverage	0.79837					
Solution consistency	0.819968					

*“•” means the condition exists*, “⊗” *means the condition does not exist, and the blank space means the condition can either exist or not; the core condition is marked with a large* “•” *and a large* “(⊗).”

For raw coverage, the six paths were 0.38, 0.53, 0.26, 0.33, 0.29, and 0.28, respectively. It can be seen that the second path has a high explanatory degree, while the other five paths show little difference in explaining the establishment factors of new ventures. According to Table 1, the core paths formed by simplified solutions can be roughly divided into three types, as shown in Table 5.

**TABLE 5:** Regional distribution of entrepreneurial core elements path.

Core path	Type	Areal distribution
1	EP + GA	GBA
2	EP + ME	GBA, Singapore, Malaysia, Indonesia, Thailand, and Vietnam
3	ME + EPY + GA	Philippines, Laos, Cambodia, Myanmar, Brunei, and East Timor

### Path Analysis for Guangdong, Hong Kong, and Macau

Table 5 shows that the core elements of new ventures in the GBA include EP, ME, and regional advantages. After the marginal conditions are added in combination with the path distribution map in Table 4, three paths can be obtained for the GBA, namely, EP + ME, EP + PA + regional advantage, and EP + CE + EPY.

The first path is EP + ME. On the one hand, the market environment is reflected in the good business environment of the GBA, such as the gradual change of business registration procedures, industrial assistance policies, and tax relief. On the other hand, Guangdong, Hong Kong, and Macao have the characteristics of a high level of economic development, large capital volume, a good financing environment, and diversified financing methods. When entrepreneurs have a good spirit, and when from a micro perspective, the market environment of the industry in which the enterprise is located has a good performance, the enterprise in the entrepreneurial process already has all the elements of this path and can rely on the market in the GBA Entrepreneurship for the environment. The second path is EP + PA + GA. On one hand, under the open and inclusive economic system of the GBA, products have strong advantages and competitiveness in market surveys, which are the material basis for the establishment of new ventures; on the other hand, having product advantages will help the company stand out in the fierce market competition and receive continuous injections of capital or technology that ensure the survival and development of the startups. Under the favorable geographical location of the GBA, positive psychological factors and product advantages will play a catalytic role and amplify the competitiveness and viability of enterprises in the initial stage.

Finally, the third path is EP + CE + EPY; while the importance of EP is important; CE as the core condition is also an important part of creating an entrepreneurial atmosphere, such as the famous “Zhejiang Merchants” culture, and the GBA has a long-standing entrepreneurial culture. As a coastal area, people have an ocean factor that leads them to dare to take risks and the hardworking spirit of small businessmen and hawkers in their bones from working hard. This spirit helps entrepreneurs stick to their goals and maintain their passion. In addition, GBA has comprehensive entrepreneurial policies for first-time entrepreneurs. This path shows that in the GBA when entrepreneurs’ startups are relatively weak in various aspects, the foundation of the regional entrepreneurial culture is influenced by government policies. Public support and assistance are essential for the establishment of new ventures.

### Southeast Asia Path Analysis

In Southeast Asia, there are two ways to establish new enterprises: EP + ME + CE and ME + EPY + GA. The first path is suitable for Singapore, Malaysia, Indonesia, Thailand, Vietnam, and other countries. Singapore and Malaysia already have perfect and open MEs based on their developed service industries. Although Indonesia, Thailand, and Vietnam lack multi-channel financing and developed economic environments, they have huge market potential and demand gaps in many industries that can greatly arouse the interest of entrepreneurs. In addition, Indonesia, Thailand, and Vietnam have a long history and cultural precipitation, which plays a supporting role in the entrepreneurship and development of enterprises and has a positive influence on the psychology of entrepreneurs. Therefore, the EP + ME + CE path applies to these Southeast Asian countries.

### Comparison of the Path Analysis Between Greater Bay Area and Southeast Asia

In addition, the Philippines, Laos, Cambodia, Myanmar, Brunei, and East Timor also show the second path. These characteristics of southeast Asian nations constitute a huge market potential, and the demand gap and regional advantage indicate that a certain area, whether in the process of its economic development, nature, economy, technology, management, or social factors, has special advantages in one or several factors that make the region more competitive or have higher resource utilization efficiency. Typical regional advantages of Southeast Asian nations are low labor costs and greater economic potential with the arrival of demographic dividends. In these countries, due to the lack of objective environment and condition, and other business aspects, the government provides policy support for startups through one of the most dangerous periods. This is also one of the core elements that southeast Asian entrepreneurs can rely on.

The path analysis results in Table 4 show that the consistency of solutions is 0.819968, greater than 0.8, the minimum consistency standard, and the coverage of solutions is 0.79837, indicating that the six paths explain the psychology of entrepreneurs and other factors affecting entrepreneurial enterprises in nearly 80% of the GBA and Southeast Asian countries. Table 5 shows that the core factors of entrepreneurship in Guangdong, Hong Kong, Macao, and Southeast Asian countries are EP, ME, and EPY.

To understand these core factors from the commonalities between the GBA and Southeast Asia, under the opportunity of economic globalization, due to the fit of geographical location and development strategy, we note that the GBA and Southeast Asia are actively engaged in the capital, technology, and services. The two places have a good market environment and potential for emerging industries, such as financial technology and biotechnology. The sound entrepreneurial policy avoids risks in the initial stage of enterprise establishment and lays the necessary foundation for enterprise development. Therefore, from the perspective of these two points, the common entrepreneurial path of the two places is entrepreneurial psychology, market environment, and geographical advantages. The difference between GBA and Southeast Asia is that the economic environment and good entrepreneurial culture in GBA can create more diversified entrepreneurial paths for entrepreneurs, and the good ME affords them the opportunity of good financing channels and capital environment that reduces the pressure on entrepreneurs to seek capital sources, enhancing the anti-stress ability of enterprises; and there is a strong entrepreneurial culture in GBA The strong entrepreneurial culture in Guangdong, Hong Kong, and Macau provides entrepreneurs with good startups ideas and views of the entrepreneurial process, which is conducive to enhancing the divergent thinking of entrepreneurs and improving their number as well as distribution, which also lays the foundation for establishing a better business environment; in terms of entrepreneurial policies, Guangdong, Hong Kong, and Macau have always had a strong entrepreneurial focus, and every year there are entrepreneurial support policies for university graduates, social workers, foreigners, and other groups, which are released by the local Human Resources and Social Security Bureau for local entrepreneurs to inquire and refer to, so the entrepreneurship policy as the core element for entrepreneurs has its own rationality.

As for Southeast Asia, the main differences between the more developed regions such as Singapore, Malaysia, Indonesia, Thailand, and Vietnam and the GBA are the different entrepreneurial cultures and development models and differences in entrepreneurial policies. In other economically underdeveloped areas in Southeast Asia, the choice of entrepreneurial roads is often limited. Although these countries have huge potential in terms of their geographical advantages and market environment, their development level is not good due to national policy mistakes and a lack of correct strategies. For example, Myanmar’s economy is shut down because of civil wars and government decision-making errors. Therefore, EP is more urgent for entrepreneurs in Southeast Asia than for those in Guangdong, Hong Kong, and Macau. Only good national policy support can give full play to the market and geographical advantages of these countries and provide real help to entrepreneurs.

### Case Study Analysis

In order to further confirm and improve the path results analyzed by fsQCA, this study uses multiple case analysis methods to further explore the commonality of new ventures in the four representative industries in the GBA and Southeast Asia. As the problem studied in this paper is exploratory in nature, such a case study helps the researcher to enter into the problem in greater depth and analyze the problem with abundant data. Since many companies in related industries in Southeast Asia are not yet listed, it is also more difficult to collect quantitative data from the education and cultural tourism industries. Selecting a classic case comparative analysis can allow the researcher to better advise investors; moreover, case studies help researchers find their way through complex phenomena, capture their essence, and compare and analyze companies more appropriately.

### Case Selection and Data Collection

By selecting a total of 46 representative startups in four industries, namely, the financial technology, biotechnology, education, and cultural tourism, from GBA and Southeast Asia, this study examines the establishment elements as well as the differences and correlations between startups in GBA and Southeast Asia. The sample selected for this study was typical, comparative, and rich. The selection criteria are as follows:

#### Typicality

This study selects representative cases such as the origin, financing status, product characteristics, and development status of the enterprises.

#### Contrast

We categorized the four industries of financial technology, biotechnology, education, and cultural tourism in GBA and Southeast Asia according to the conditions for establishing new ventures, and compared and analyzed similar cases. Richness: The selected cases involve various industries and regions, and each of the typical cases in the GBA and Southeast Asia has the typical characteristics of new startups, which is in line with the theme of this article. Based on the above criteria, this study selected 46 new ventures in four popular industries for typical case studies (as shown in Table 6).

**TABLE 6:** The commonality of each of the four industries in the two regions.

Industry	GBA	Southeast Asia
Financial Technology	1. A good cultural background can to a large extent form the psychological tone of entrepreneurs, and even in cross-border trade and study abroad, values and psychological trends that are compatible with the local culture will be implicitly formed.	1. New ventures in the financial technology industry are mainly concentrated in economically developed regions with good industry support policies such as Singapore and Malaysia.
Education	There is a strong entrepreneurial cultural atmosphere and a good market environment in education entrepreneurship. The Ministry of Education, Development and Reform Commission and other government departments also support education industry policies. 2. The double reduction policy issued by the General Office of the State Council on July 24, 2021, is a major blow to online education.	1. Southeast Asia has a lack of educational resources and a poor entrepreneurial environment, but the local market environment and the demand for education is vast. 2. Online education is developing rapidly under the epidemic.
Biotechnology	1. GBA Bay Area is an important region for China’s biotechnology and pharmaceutical industries, providing a good entrepreneurial ecological environment for startups.	1. The Singapore government provides financial support for biotechnology and pharmaceutical R&D, with an annual R&D investment of S$1.5 billion and tax incentives. 2. The governments of a few Southeast Asian countries, such as Singapore and Thailand, provide financial support and tax incentives for the research and development of biotechnology medicines every year.
Cultural Tourism IP	1. GBA has a good atmosphere of cultural tourism entrepreneurship and a strong cultural heritage. 2. Cultural and tourism startups are organically combined with local characteristics and folk customs to shape cultural and tourism IP.	1. Southeast Asia has a long cultural history, and it is also a popular destination for Chinese tourists traveling abroad. It has a good entrepreneurial atmosphere. 2. Southeast Asian countries with developed tourism industries such as Malaysia and Indonesia have introduced a number of policies to help the development of cultural tourism.

The information for this paper was mainly obtained from the public information of each company’s official website, news reports obtained by searching the latest developments of national or regional statistical bureaus as keywords, and tweets from several public websites, in addition to studying several reports, including line research reports; relevant data were mainly obtained from the World Bank database, China Statistical Yearbook, and the websites of major research institutes.

**FIGURE 1:**
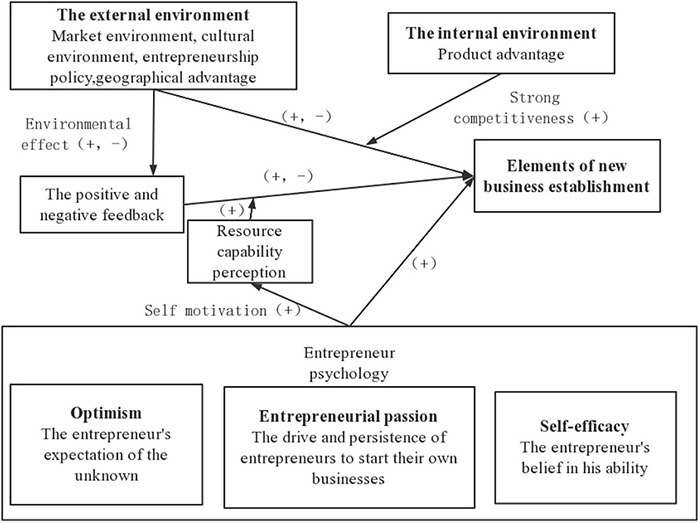
Structure of the elements for the establishments of startups. “(+)” means positive effect, “(–)” means negative effect, “(+,–)” means may have a positive or negative effect.

The current factor structure of startups is shown in Figure 1, in view of the difficulties faced by entrepreneurs in setting up enterprises, particularly the negative impact of adverse environments. We took the entrepreneur psychology (optimism, entrepreneurial passion, self-efficacy) as the core of the elements of startups to perceive the environment, which find a positive impact on entrepreneurial behavior. We got feedback from the change of external environment (market environment, cultural environment, entrepreneurship policy, regional advantage), focusing on adapting to the external environment of development and building a perfect internal environment (product advantage) according to the feedback, rather than choosing to stay away from the bad environment and giving up the existing market. The above analysis reveals that the reasonable use of entrepreneurial factors can help enterprises avoided difficulties and improved survival rate and anti-risk ability.

From Table 5, we can see that the main motivations for six fintech startups to flourish in both GBA and Southeast Asia are inseparable from the strong economic strength and advanced technology of GBA, as well as the geographical advantage and demographic dividend of the Southeast Asian market. In addition, the Internet penetration rate and the ease of doing business in Southeast Asia have also influenced the combination of factors for the establishment of new startups in both regions. For example, in terms of entrepreneurship policies, Singapore and Malaysia are rapidly developing entrepreneurship policies for foreign populations, the Indonesian government has proposed “6 + 5” 11-year compulsory education in the education sector, and the Vietnamese government has proposed increasing investment in the cultural tourism industry, encouraging investment, simplifying tourism procedures, and giving a green light to tourism development. The combination of various factors will have different effects on promoting entrepreneurship in different industries.

The current case study conducted in Table 5 focuses on the following questions:

### Comparison of the Elements of New Startups Among Different Regions in Greater Bay Area and Southeast Asia

First of all, from the perspective of the size of enterprises, the enterprises in the GBA in the biotechnology, education, and cultural tourism industries are generally larger than those in Southeast Asia. Half of the new startups in Southeast Asia in these industries are SMEs that are not listed because the business environment of these industries in the GBA is better than that in Southeast Asia, including richer financing channels and more open ways of absorbing funds, providing objective conditions for the establishment of new ventures; second, the current development of the cultural environment and entrepreneurial policies in GBA are better than those in Southeast Asia in terms of cultural environment and entrepreneurial policies, especially in the cultural tourism industry, relying on the long tradition of cultural heritage and government entrepreneurial policy support; Guangdong, Hong Kong, and Macau have achieved fruitful results in the development of cultural tourism intellectual property (IP). The cultural tourism IP industry of Indonesia, Malaysia, Vietnam, and other countries in Southeast Asia has a profound cultural heritage, precious historical relics, and development issues. This is mainly due to the excessive dependence on consumer culture, history, and tourism. The development of the cultural and tourism industry is largely restricted by the number of tourists and seasonal changes. On the other hand, as the business model is not novel enough and government policy support is small, it is difficult for cultural and tourism IP creators to obtain the necessary income for survival. In general, in terms of the region, due to the degree of economic development, differences in financing environment, and different cultural atmospheres, GBA has comparative advantages over Southeast Asia in terms of market environment, cultural environment, and government policy elements. Therefore, some new startups in Southeast Asia are small in size, slow in development, and difficult to maintain.

### Comparison of New Venture Elements Among the Four Industries

Through comparison, we can find that the financial technology industry, an emerging high-tech industry, is usually concentrated in Singapore and Malaysia, more developed regions in Southeast Asia, and the entrepreneurial policies in Singapore and Malaysia are quite sound, which shows that the financial technology industry has a high demand for the market environment and policy support. The biotechnology industry requires a smooth flow of technology and capital elements, and is not strictly related to market requirements; the education and cultural tourism industries are also popular and well developed in Southeast Asian countries, which is mainly the result of the combined effect of their geographical advantages and market environment. With the general emphasis on education, online education institutions are becoming cheaper to start up, and the advent of the COVID-19 pandemic has made the prospects of online education extremely broad. Southeast Asia has a large education population potential due to the population dividend that has not yet arrived, which is a geographical advantage, and new startups in the cultural tourism industry rely on the convenience of traditional culture and the market environment in some countries where certain religious beliefs flourish.

### Comparison of Importance and Applicability Among Different Elements

From the perspective of the elements, entrepreneur psychology is the most important factor affecting the entrepreneurial process. Among other factors, the cultural environment has the greatest impact on entrepreneur psychology, and regional differences will have different decision-making influences and potential influences on entrepreneur psychology. Second, regarding the market environment and cultural environment, the market environment of good regions and industries can create a good financing environment and sufficient sources of funds for enterprises, while a good cultural background can, to a large extent, form the psychological tone of entrepreneurs, and even in cross-border trade and study abroad, values and psychological trends that are compatible with the local culture will be implicitly formed. Finally, regarding entrepreneurial policy, geographical advantage, and product advantage, entrepreneurial policies are the government’s help and support for entrepreneurs; the more economically developed regions generally have a comprehensive entrepreneurial policy covering different groups of the entrepreneurial population that can provide comprehensive support and subsidies for entrepreneurs, while less developed regions in Southeast Asia lack an effective or strong entrepreneurial policy. An example is Malaysia and Indonesia’s cultural tourism industry policy, which has difficulties making up for the real situation of low salaries of industry entrepreneurs. The geographical advantage of Southeast Asia is its huge market development potential and population potential, so most Southeast Asian startups may create new businesses based on this to promote development. Finally, product advantage depends on the existence of the other factors, because if multiple conditions are not ideal, it is difficult to generate competitive product advantages, which will subsequently lead to the poor market performance of startups and difficulties in survival and expansion.

In addition, we have also noted that no region can be regarded as the same in terms of cultural environment. For example, Southeast Asian countries, Indonesia, Malaysia, Vietnam, and other countries have their unique cultural deposits, which subtly affect entrepreneurs’ entrepreneurial spirit. The characteristics of social culture not only have an impact on the entrepreneurial process of entrepreneurs but also have a huge impact on entrepreneurs in different regions (Vuong, 2016). Among them, religions, folk customs, and other historically embedded cultural elements largely determine the complexity of entrepreneurs’ decisions (Vuong et al., 2020). Even if they receive education in other countries or regions, they will also be influenced by the local society and culture, and their values will change accordingly, which can easily occur in the context of geographical migration or cross-regional communication common among entrepreneurs. Based on the special value and influence of the social culture mentioned above, we consider the influence of cultural factors in regions with distinctive cultures in the output of the follow-up path results.

In the actual interview, we asked nine start-up entrepreneurs from GBA, Singapore, and Vietnam about entrepreneurship, which are the biggest difficulties in entrepreneurship, the impact of local culture on decision-making, and the factors of industry choice. In the difficulties faced by entrepreneurship, most entrepreneurs believe that compared with technical difficulties and product problems, whether to have strong psychological quality is the watershed of entrepreneurial failure, which is also the primary reason why many young people fail to start a business: cannot adhere to entrepreneurial behavior, as an investment in their own. As for the impact of local culture on entrepreneurial decision-making, entrepreneurs have different views, some entrepreneurs believe that in these transnational cultures, although promoting the public’s willingness to start a business, but too impetuous ideas will affect the next step of cooperation with partners or the government, and entrepreneurs believe that local culture plays an important role in stimulating entrepreneurial decision-making, but cannot make up for the lack of entrepreneurial capacity, because of the need to strengthen the linkage between entrepreneurship and educational psychology. In the choice of industry, some entrepreneurs think that is the result of their own educational experience and market environment, but some entrepreneurs think that they benefit from their long-term strategic vision.

### Case Summary

Through the analysis and research comparison of some of the startups that represent the new economic format in the GBA and Southeast Asia, we find that once these startups become unicorns, they will inevitably increase the economic and development potential of an industry. Greater enhancement often leads to technological progress and industrial upgrading. This kind of start-up enterprise is divided into two types of teams: a technical team and a commercialization team. The technical team mainly focuses on the planning and application of patented technology, followed by product design and technology application. The commercialization team mainly focuses on the planning and execution of the company’s operation plan, as well as the marketing promotion, product application, and other aspects. The survey found that policies have a positive incentive effect on enterprises. For example, the Outline Development Plan for GBA policy implemented by the GBA has given support to the development of local financial technology, biotechnology, education, cultural and tourism IP, and other industries. Sustained support, such as the “S$1.5 billion” support plan implemented by Singapore in Southeast Asia, has also provided conditions for the initial survival and development of new startups.

**FIGURE 2:**
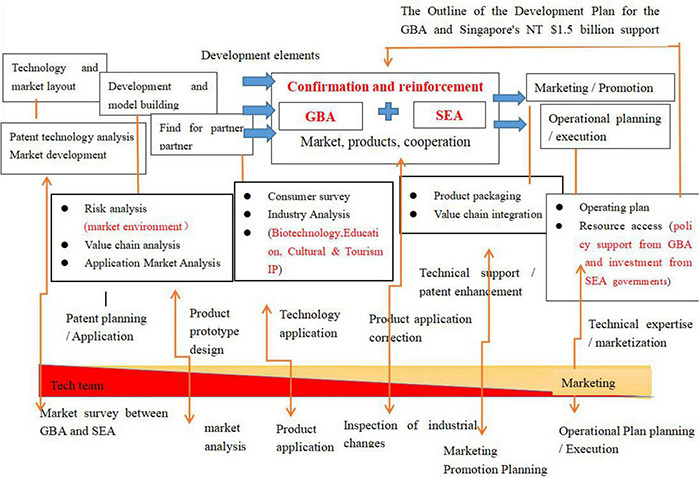
Dynamic teamwork path evolution from idea to market of startup.

As shown in Figure 2, the factors of product advantage, market environment, and regional advantage mainly affect the technical teams of new enterprises in patent applications, product prototype design, technology application, and other aspects. These factors could confirm and strengthen an enterprise’s development elements, such as technology, market layout, R&D, model building, and finding partner manufacturers.

The advantages of the cultural environment and entrepreneurial policies mainly affect the implementation of the operation plan and marketing promotion of the commercialization team, enabling them to obtain the support resources of local policies and the integration of the industrial chain, so that the product can be better operated and promoted.

Under the joint action of the five factors, the technology and commercialization development of enterprises will be improved, having a positive impact on entrepreneurs.

## Results

To explore the configuration effect of the influence of entrepreneurial factors on the establishment of new ventures, this study uses a questionnaire survey to survey 46 representative new ventures from four industries in GBA and Southeast Asia using qualitative comparative analysis. fsQCA explores the path combinations that play a key role in the establishment of new ventures from the six factors of entrepreneurial psychology, product advantages, market environment, cultural environment, entrepreneurial policies, and geographical advantages, and further uses specific industry cases to verify the path combination. Reasonability and credibility, the main conclusions are as follows:

(1) From Table 7 we can find five combined paths affect the establishment of new ventures: “psychology product and region,” “psychology, culture, and policy,” “psychology and region,” “psychology and market,” and “market and policy.” The original coverage rates of the five paths have a certain gap. The coverage rate of psychological and regional paths in the GBA and Southeast Asia is as high as 0.53, which is much higher than other paths, indicating that this path has strong explanatory power. The coverages of the other four paths show a little gap, and the difference in the effect of improving the establishment of new ventures is small. Based on this, each region can promote the psychological cultivation and publicity of innovators according to the *status quo* of the entrepreneurial environment and increase the product advantages of new startups. At the same time, a reasonable path for regional entrepreneurship can be selected based on economic development.

**TABLE 7:** Distribution of factors for establishing cross-regional and multi-industry startups.

Paths	Industry	Region
EP + PA	FT + BT	GBA
EP + CE + EPY	EI + CT	GBA, Indonesia, Malaysia, and Vietnam
EP + GA	BT + CT	SEA
ME + EPY	EI + CT	Philippines, Laos, Cambodia, Myanmar, Brunei, and East Timor
EP + ME	FT + EI	GBA, Singapore, Malaysia, and Indonesia

(2) Entrepreneur psychology, market environment, and entrepreneurial policies are the core conditions for improving the effectiveness of the establishment of new ventures. The remaining three variables are non-core variables in different paths. The decision-making of a new venture is largely influenced by entrepreneurial psychology. A good market environment and entrepreneurial policy support are sufficient conditions for the establishment of new ventures. The three core elements are synergized, coupled with the assistance of various non-core elements, and have a good effect on promoting the establishment of new ventures. Therefore, no matter which path a given region chooses, it should firmly grasp the core elements and comprehensively improve the entrepreneurial environment.
(3) Different regions or industries have different paths for entrepreneurship and factor combinations. We find that factors in the GBA are relatively complete and balanced, offering enterprises a choice of multiple paths in the establishment process, which has obvious advantages in promoting entrepreneurship; financial technology (FT) and biology technology (BT) industries are more dependent on their product advantages and the market environment in the more developed regions such as the GBA, Singapore, and Malaysia. The education, cultural, and tourism industries are affected to a certain extent by the local cultural heritage, but for most of the Southeast Asian countries, startups are more inclined to market potential and entrepreneurial policy assistance.

## Suggestions

### According to Local Conditions, Choose the Driving Path Reasonably

The advantage of the fsQCA method is that it can identify the sample area represented by a specific path and combine the specific area to summarize and compare the promotion path of new ventures; thus, the conclusions are more targeted and realistic. Therefore, based on the scientific evaluation of the entrepreneurial environment of new startups, each region should explore new startup paths that suit the actual conditions of each region. First of all, for the GBA, Singapore, Malaysia, and other places where the entrepreneurial environment (market environment, entrepreneurial culture, etc.) has developed in a balanced manner, multiple paths can be selected to enhance the survival of new startups and comprehensively improve the entrepreneurial environment. Regions with a more specific entrepreneurial environment, such as Vietnam and Indonesia, can choose the driving path with the highest matching degree. For example, Vietnam can choose the combination path of “psychology, culture, and policy” and Indonesia can choose the combination path of “psychology and market”; finally, for economically underdeveloped areas with a general entrepreneurial environment (GBA and some countries and cities in Southeast Asia), such areas need to scientifically evaluate the development level and interaction of each element of entrepreneurship, and choose a suitable and highly matched driving path. The effect of establishing new ventures can be improved through “curving overtaking,” such as the development of education and cultural tourism industries.

### Focus on Key Points and Implement Precise Policies for Key Elements

All regions should focus on the key elements that can effectively improve the survival of new startups, improvements in the entrepreneurial environment and policy service system, and the cultivation and promotion of entrepreneurial positive psychology. First, they should work on improving the entrepreneurial environment, increasing market financial support based on the characteristics of new ventures, raising the share and proportion of government fiscal funds in the policy and capital tilt during the establishment of new ventures, and effectively guiding new ventures onto the right track. Second, they should optimize the policy service system. The government should improve the efficiency of management services, truly provide a “one-stop” service for startups, further integrate regional entrepreneurial resources by optimizing entrepreneurial policies, promote the diffusion of information and knowledge sharing between the government and startups, and obtain innovative technologies for entrepreneurs: Product information, entrepreneurial experience, and management knowledge constitute effective channels for this; finally, they must pay attention to the cultivation and publicity of entrepreneurial psychology. Regional entrepreneurial psychology must be multi-pronged to establish a positive entrepreneurial value system, such as establishing new entrepreneurial benchmarks or enterprises. An entrepreneurial brand should be created, full use made of the brand effect, and the success stories of new ventures actively spread to improve the public’s entrepreneurial awareness, create a tolerant entrepreneurial atmosphere, make full use of the psychological elements of entrepreneurship, increase positive and positive entrepreneurial psychological guidance, and improve entrepreneurial efficiency. By making full use of the psychological elements of entrepreneurship, governments can increase the positive psychological guidance of entrepreneurship and improve the efficiency of entrepreneurship.

### Integrate Resources and Contribute to the Synergistic Effect of Various Entrepreneurial Elements

The key to improving the survival of new ventures lies in the interaction of different entrepreneurial elements to form a synergy. Therefore, different elements must be effectively integrated to enhance synergistic effects. First, it is necessary to establish effective coordination, linkage, and unified management service mechanisms at the government level, and strengthen the in-depth integration and optimal allocation of elements of the entrepreneurial environment. All regions must coordinate to build an entrepreneurial environment and service guarantee system that conforms to the laws of entrepreneurship and the market economy, guides new ventures to take root, and improves the survival of new ventures. Second, each region can use policy tools to improve the entrepreneurial environment. These include the detailed implementation of policy arrangements for new ventures, establishing and improving local support policies to promote new ventures, guiding industrial capital and venture capital to participate in corporate financing, increasing special support funds for new ventures, improving the entrepreneurial market operation mechanism, and strengthening the foundation for innovation and entrepreneurship facilities construction.

There are three limitations of this article that should be borne in mind. First, as some of the studied enterprises in Southeast Asia are not listed and the information used in data collection is limited, in-depth data collection should be carried out in the future to identify classic and effective cases. There is also a certain correlation between the psychological factors of entrepreneurs and other elements of the establishment of new ventures, so they can be studied from the interactive effects of multiple factors in the future. Finally, the static data used in this study and the dynamic data of entrepreneurs in the process of establishing enterprises can be collected for analysis in the future, which can improve the credibility and scientific nature of the research results.

## Data Availability Statement

The original contributions presented in the study are included in the article/Supplementary Material, further inquiries can be directed to the corresponding author/s.

## Author Contributions

XW and C-CC: conceptualization, methodology, and software. Z-HZ: data curation and writing – original draft preparation. YT and HW: regression and writing – original draft preparation. ZC: writing – reviewing and editing. All authors contributed to the article and approved the submitted version.

## Conflict of Interest

The authors declare that the research was conducted in the absence of any commercial or financial relationships that could be construed as a potential conflict of interest.

## Publisher’s Note

All claims expressed in this article are solely those of the authors and do not necessarily represent those of their affiliated organizations, or those of the publisher, the editors and the reviewers. Any product that may be evaluated in this article, or claim that may be made by its manufacturer, is not guaranteed or endorsed by the publisher.
