# Supplementary material for: The Analysis of Fuzzy Qualitative Comparison Method and Multiple Case Study of Entrepreneurial Environment and Entrepreneur Psychology for Startups—Evidence From Guangdong-Hong Kong-Macao Greater Bay Area and Southeast Asia
Source: Front Psychol. 2022 Jan 25;12:751309. doi: 10.3389/fpsyg.2021.751309 (PMC8821655; doi:10.3389/fpsyg.2021.751309)
Supplement: Supplementary file 1 [file Table_1.docx]

| Enterprise | Products | Market | Policy | Business |
| --- | --- | --- | --- | --- |
| RazerPay(Singapore) | Singapore and Thailand are currently working together to develop the world's first real-time cross-border agent payment system.  Singapore has shifted from cash payments to cardless payments, joining the arena of the electronic payments market. | Singapore is at the forefront of digital payments in Southeast Asia and was one of the first countries in Southeast Asia to introduce payment agent payments, with a very educated population and high per capita income and high consumer demand. | Implementing grants, tax incentives and in-kind assistance programs to attract more business investment in fintech. | RazerPay is currently one of the largest e-wallets in Malaysia；  RazerPay joins hands with NETS to operate Singapore's e-payment market；  RazePay launches offline and online electronic payment services. |
| Boost | UnionPay International has partnered with Malaysia's Axiata Telecom Group to enable its wallet product Boost to support UnionPay's QR code payment service through electronic card issuance. | Malaysia:  Total population 32.66 million (2019).  Total GDP of US$358.582 billion.  GDP per capita of US$11,200,000.  25.84 million Internet users.  Internet penetration rate of 80%. | Malaysia has further liberalized its financial sector, reduced taxes, lowered minimum investment requirements, and strengthened its integration with the international economy. | Boost is one of the most popular e-wallets in Malaysia. |
| Amartha | Amartha focuses on providing microfinance services to women micro-entrepreneurs in rural areas and mentoring the unbanked living below the poverty line. | Indonesia ranks fourth in the world in terms of total population, and Internet users are increasing year by year. As the consumption level and desire of Indonesian nationals continue to rise, the demand for payment experience will also increase. | Propose policies to encourage fintech companies to innovate and test the market, revise POJK regulations and increase supervision. | Amartha offers lending services as the peer-to-peer lending platform of amartha.com. |
| WeChat Bank | WeChat Bank has successively launched products such as Microfinance [8], Micro Industry Loan, Micro Car Loan, WeChat Bank App, WeChat Enterprise Aipu App, Little Goose Flower Money, and We2000 [9], which have served more than 250 million individual customers and over 1.7 million corporate customers. | In 2020, the GDP of the financial sector in the "9+2" Guangdong-Hong Kong-Macao Greater Bay Area will reach 1.5 trillion yuan, accounting for 12% of GDP, significantly higher than the national average of 8%. In recent years, the Hong Kong Stock Exchange has continued to improve the interconnection platform connecting the two markets, attracting a large number of new economy companies from the Mainland to list in Hong Kong, which is the most dynamic and high-growth in the world.  In 2020, the GDP of the financial sector in the "9+2" Guangdong-Hong Kong-Macao Greater Bay Area will reach 1.5 trillion yuan, accounting for 12% of GDP, significantly higher than the national average of 8%. In recent years, the HKEx has continued to improve the interoperability platform connecting the two markets, attracting a large number of new economy companies from the Mainland to list in Hong Kong, making it the most dynamic high-growth emerging market in the world.  Translated with www.DeepL.com/Translator (free version) | The implementation of the "10 financial technology" to enhance financial services to the real economy and science and technology innovation capacity, around the enterprise settlement, development and growth, application scenarios, industrial gathering, ecological building and other levels of support. | Wezubank received its financial license issued by Shenzhen Banking Regulatory Bureau in December 2014 and is the first private bank to open in China. Wezubank strictly abides by national financial laws and regulations and regulatory policies, and is committed to providing differentiated, distinctive, high-quality and convenient financial services to the general public and micro and small enterprises based on compliant operation and sound development.  Translated with www.DeepL.com/Translator (free version) |
| Sugiyama Data | Sugawan Data has now launched storage products for blocks, objects, and files, as well as encrypted secure storage products. |  |  | The main mission of Shenzhen Sugiyam Data Technology Co., Ltd. is to provide big data intelligent storage products and solutions. |
| E-Link Cloud | It mainly provides technology development, technology promotion, technology transfer, technology consulting and technology services; sales of computer software; basic software services; application software services; software development; product design; model design; design, production, agency and release of advertisements, etc. |  |  | Linklogis (English name: Linklogis) responds to the national call for financial inclusion and focuses on the application of ABCD (AI, blockchain, cloud computing, big data) and other advanced technologies in the supply chain ecology. Through customized solutions and industry-leading technology, we can bring tangible value to all parties in the supply chain finance ecosystem. |

| Enterprise | Region | Culture | Products | Market | Policy | Industry |
| --- | --- | --- | --- | --- | --- | --- |
| Junming Group | Foshan Gaoming Junming City | The cultural vein of GBA Greater Bay Area refers to the most splendid and remarkable life undercurrents in the development of this place for thousands of years. It does not come from elsewhere, but from the confluence of geography, history and ethnicity, and it is the lifeblood that supports the development of the region and is the spirit and soul of a region.  From the perspective of the cultural and historical accumulation and development of the Guangdong-Hong Kong-Macao Greater Bay Area, the Guangdong-Hong Kong-Macao Greater Bay Area as a whole is more traditional than traditional and more modern than modern. | 《Meet in Foshan》 | By 2020, Guangdong Province will receive a total of more than 500 million overnight visitors, with total tourism investment of RMB 200 billion and total tourism revenue of RMB 1.6 trillion. The comprehensive contribution of tourism to the national economy of Guangdong reaches 13%.  In the tourism segment of Guangdong-Hong Kong-Macao Greater Bay Area, there are many super tourism IPs gathered: Hong Kong Disneyland, The Venetian Macao, Guangzhou Guangzhou Changlong Wildlife World, etc. Guangdong-Hong Kong-Macao will also become the region with the most super tourism IPs in China. | The announcement of 《the Announcement on Tax Policies Related to the Prevention and Control of the Pneumonia Epidemic in Support of Novel Coronavirus Infection》 states that the four major categories of difficult industry enterprises in transportation, catering, accommodation and tourism (referring to travel agencies and related services, tour scenic area management in two categories), which are more affected by the epidemic, have the longest carry-forward period from 5 years to 8 years for losses incurred in 2020. | In the Guangdong-Hong Kong-Macao Greater Bay Area tourism industry continues to drive, for the cultural and creative driven travel service products are standing in the way of consumer upgrading, behind the development of cultural and creative industries in GBA support, the prospects are clear. |
| Foshan Sanshui Sunlake Tourism Co. | Sanxiang Village, Baini Cultural and Creative Ancient Town, Sanshui District, Foshan City |  | China's first lacquer art village |  |  |  |
| Guangdong Travel Creation Culture and Creativity Co. | Guangzhou Shawan Ancient Town |  | Guangzhou Shawan Ancient Town Original IP |  |  |  |
| AKSI (Asosiasi Komik Indonesia) | Malaysia | The cultural region of Southeast Asia is mainly influenced by India and China, but each has a very distinctive personality, and its art and architecture are significantly different from those of India and China. | Movie, Comic IP | According to the 《Report》, more than half of the animation companies in Southeast Asia are now established in the last 6 years, with the largest number of companies established 4~6 years ago, accounting for 28%. | The National Tourism Policy 2020-2030 was launched to encourage investment in technology-based tourism and to support the development of tourism businesses. | In Southeast Asia, only 33% of animation companies have their own animation IPs, and a significant portion of animation companies are living on orders for animation production, while the salaries of those working in animation are extremely low. For example, the approximate monthly salary in the Philippines is only between Php13,000 ($1790) and Php16,000 ($2200). |
| KING Productions | Indonesia |  | Comic IP |  | Promote Tourism 4.0 policies to improve natural, human and cultural productivity and encourage and support business innovation. |  |
| VinTaTa Animation | Vietnam |  | Comic IP |  | increasing the investment power of the Vietnamese government in the cultural and tourism sector to encourage investment  Simplify tourism processing procedures and give the green light to the development of tourism. |  |

| Enterprises | Products | Market Environment | Policy | Business Type |
| --- | --- | --- | --- | --- |
| Palm Education | Online customized education platform, Tsinghua University Psychology Center will provide academic support for the online quality education institute, Palm Education has joined hands with a number of national platforms such as Learning Power, Xinhua News Agency and Central Video to open a large number of live courses for free, covering all school age groups. | There is a huge demand for education in China, and the need for online education not only exists in the K12 field, but also extends to vocational education, professional education and many other areas, and the potential users of online education are huge. | April 2021, the National People's Congress issued the Education Law of the People's Republic of China (2021 Amendment), proposing to develop and attach importance to education.  In January 2021, the Ministry of Education, the Development and Reform Commission and the Ministry of Industry and Information Technology proposed to make the integration and application of information technology in education teaching a major strategic project to promote "education + Internet", deepen the reform of the way of educating people in basic education and accelerate the modernization of education, strengthen systematic planning and intensify work.  In September 2019, the Ministry of Education, the Central Internet Information Office and the Development and Reform Commission issued a guiding opinion on promoting the healthy development of online education, requiring the full use of modern information technology to provide online education services. | All-disciplinary education and comprehensive education. |
| Octopussy Education | "5+8+5" team of professors to develop solutions.  Innovative educational growth planning for schools.  A powerful education cloud platform, creating an intelligent software ecosystem.  "Spark" program to improve the teaching level of teachers.  500+ lessons selected strictly to create STEAM quality lessons. |  |  | Software development.  Curriculum teaching products.  Confrontation class creative manufacturing kit/provide Haiba STEAM creator education curriculum solutions. |
| Goodwill Education Group | Strong team qualifications.  High-end education brand.  Strong industry integration: integration of the relevant industries of various groups under Jia Zhaoye, strong cooperation, multi-win cooperation and maximum utilization of resources. |  |  | With high-quality international education as the mainstay, Goodwill Education Group has built a one-stop education system with linkage development of preschool and K12 education, and actively expanded community education and online education. |
| ruangguru | "Ruangguru" says that by using high-end technology, the platform publishes high-quality content for students so that millions of students can enjoy quality education. | Most Southeast Asian countries generally suffer from a lack of educational resources and uneven regional distribution, and there is a high demand for education in the market. | In 2012, the Indonesian government published the "Education Support Initiative" policy, which aims to promote higher quality development of nine-year compulsory education.  In 2004, the Department of Education introduced the vision of "teaching less and learning more". It calls for educators to teach better, engage students, and prepare them for life.  The "6+5"' 11-year free education policy, which places a high value on education. | Providing education-related services such as video subscriptions, tutoring and corporate learning, it currently has more than 15 million users and over 300,000 teachers, offering services covering more than 100 subject areas. |
| Classruum | Using the social media distribution method, users can form groups with friends and learn together online. |  |  | Features video instruction, notes, digital library, exercises, social bookmarking, one-on-one video instruction, and themed games. |
| topica | Topica Education Group is a leading online education provider in Southeast Asia. Offering high quality online degree programs, a number of accredited U.S. universities have also recognized Topica-supported programs for credit. |  |  | With many successful products including "Topica Uni", "Topica Native". |
| Coursera | Coursera is an educational platform that partners with the world's top universities and institutions to provide free online courses that anyone can take. |  |  | Universities are ushering in a new era of digital transformation that demands high-quality online learning options. |

| Enterprise | Region | Culture | Products | Market | Policy· | Industry |
| --- | --- | --- | --- | --- | --- | --- |
| Avion Pharmaceutical (Zhuhai) Co. | Hengqin New Area, Zhuhai | GBA have Lingnan culture as their common root. In recent years, the three places have attracted each other in many fields, using their respective strengths to promote the accelerated pace of the construction of the Guangdong-Hong Kong-Macao Greater Bay Area, painting new colors for the development of Lingnan culture in the new era. | Innovative eye drop iVIEW-1201 for broad-spectrum sustained release treatment of viral conjunctivitis. | According to statistics, the number of cases of viral conjunctivitis in China can reach 15 million each year. There is no drug approved by the FDA for the treatment of viral conjunctivitis worldwide. | The Outline of the Development Plan of Guangdong-Hong Kong-Macao Greater Bay Area proposes to promote the development and growth of new generation information technology, biotechnology, high-end equipment manufacturing and new materials as new pillar industries.  On July 8, 2020, the Guangdong Provincial Science and Technology Department officially issued the Notice on Several Policy Measures to Promote the Innovative Development of Biomedicine, with a total of 10 measures. The notice points out that Guangzhou and Shenzhen should be the core to create a biomedical science and technology innovation cluster in Guangzhou-Shenzhen-Hong Kong and Guangzhou-Zhuhai-Macau. And in the clearance of biologic materials, drugs and medical devices flow, etc., to open up the cross-border flow of GBA innovation factors path. | The Guangdong-Hong Kong-Macao Greater Bay Area is an important region for China's biomedical industry to gather, with a complete industrial foundation and leading strength.  The development of biomedical industry in the Guangdong-Hong Kong-Macao Greater Bay Area is moving into a new stage of integrated and collaborative development.  GBA biopharmaceutical industry has a strong foundation, high integration and strong vitality。 |
| Transcend International Biotechnology Co. | Transcend International is headquartered in Guangzhou. |  | Focusing on organoid technology development and industrialization. | There is an international shortage of organs |  |  |
| Dashi Pharmaceutical (Guangdong) Co. | Cuiheng New District, Zhongshan City |  | DS002 injection is the only known domestic monoclonal antibody drug targeting NGF. | DS002 injection is the only known domestic monoclonal antibody drug targeting NGF, which has shown significant efficacy in a variety of preclinical studies. and has been approved for clinical trials. |  |  |
| Etana Biotechnologies | Indonesia | Southeast Asia is a region with diverse unity. This, coupled with frequent ethnic migrations and cultural interactions among various ethnic groups, has resulted in a diverse pattern of life and colorful ethnic cultures. | Erythropoietin (EPO). | According to BMI, pharmaceutical sales in the ASEAN region exceeded $20 billion in 2014 and are expected to reach $40 billion by 2020. | Increase funding for biotechnology and implement a bio-incubator project support program. | Southeast Asian countries face multiple development challenges, and governments have had to engage more private providers in building the capacity of their healthcare systems. However, the private sector is mostly focused on tertiary care services and specialty care. |
| AUM Biosciences | Singapore |  | Focusing on the screening of small molecule anti-tumor innovative drugs, clinical I and II development. | The Association of Southeast Asian Nations (ASEAN) ASEAN has 10 member countries with a total population of over 654 million. Some surveys show that population aging (people over 65 years old) will gradually increase in the next 20 years, while the prevalence of chronic diseases such as diabetes and cancer is also increasing. | Implementing R&D support with an annual investment of S$1.5 billion in biomedical R&D.  Provide tax incentives to provide certain benefits to biomedical companies. |  |
| Baiya Phytopharm | Thailand |  | Tobacco leaf R&D to prepare new crown vaccine。 | In more developed countries such as Singapore and Thailand, the population over 65 years of age already exceeded 6.7% in 2018 and is expected to reach 10.8% in 2030. In this context, the population will have an increasing demand for medicine and healthcare | Implementing a series of preferential policies for investment in biotechnology, granting a three-year reduction to small and medium-sized enterprises that invest in research and development projects, and creating a medical industry hub and an export base for medical equipment. |  |
